# Screening of key miRNAs related with the differentiation of subcutaneous adipocytes and the validation of miR-133a-3p functional significance in goats

**DOI:** 10.5713/ab.22.0121

**Published:** 2022-06-30

**Authors:** Xin Li, Hao Zhang, Yong Wang, Yanyan Li, Youli Wang, Jiangjiang Zhu, Yaqiu Lin

**Affiliations:** 1 Key Laboratory of Qinghai-Tibetan Plateau Animal Genetic Resource Protection and Utilization of Ministry of Education/Sichuan Province, Southwest Minzu University, Chengdu 610041, China; 2 College of Animal and Veterinary Sciences, Southwest Minzu University, Chengdu 610041, China

**Keywords:** Adipocyte Differentiation, Goat, miRNA-seq, miR-133a-3p

## Abstract

**Objective:**

Adipocyte differentiation is regulated by a variety of functional genes and non-coding RNAs. However, the role of miRNAs in lipid deposition of goat white adipose tissue is still unclear. Therefore, this study revealed the miRNA expression profile in goat subcutaneous adipocytes by sRNA-seq.

**Methods:**

The miRNA expressed in goat subcutaneous preadipocytes and the mature adipocytes were sequenced by sRNA-seq. The differentially expressed miRNAs (DEm) were screened and gene ontology (GO) and Kyoto encyclopedia for genes and genomes (KEGG) analyses were performed. Gain-of-function and loss-of-function combined with oil red O staining, Bodipy staining, and quantitative reverse-transcription polymerase chain reaction (qPCR) were utilized to determine the effect of miR-133a-3p on adipocyte differentiation.

**Results:**

A total of 218 DEm were screened out. The target genes of these DEm were significantly enriched in GO items such as biological regulation and in KEGG terms such as FAK signaling pathway and MAPK signaling pathway. qPCR verified that the expression trend of miRNA was consistent with miRNA-seq. The gain-of-function or loss-of-function of miR-133a-3p showed that it promoted or inhibited the accumulation of lipid droplets, and CCAAT enhancer binding protein α (*C/EBPα*) and *C/EBPβ* were extremely significantly up-regulated or down-regulated respectively (p<0.01), the loss-of-function also led to a significant down-regulation of peroxisome proliferator activated receptor gamma (*PPARγ*) (p<0.01).

**Conclusion:**

This study successfully identified miRNAs expression patterns in goat subcutaneous adipocytes, and functional identification indicates that miR-133a-3p is a positive regulator of the differentiation process of goat subcutaneous adipocytes. Our results lay the foundation for the molecular mechanism of lipid deposition in meat-source goats from the perspective of miRNA.

## INTRODUCTION

Goats as domesticated animals are one of the main sources of meat [1]. Compared with beef and pork, chevon has a unique flavor and is widely welcomed by consumers in China. Mutton flavor substances are mainly accumulated in adipose tissue [2], adipocyte is the main component of adipose tissue and adipocyte differentiation is the key process of adipose deposition. Studies have shown that the differentiation of animal adipocytes is regulated not only by functional genes but also by non-coding RNAs such as miRNAs [3].

MicroRNAs are a class of endogenous non-coding RNA about 22nt and are widely found in plants and animals [4]. In contrast with plants, miRNAs in animals can cleave or suppress translation by binding to target mRNAs that are not completely complementary, thereby playing an important regulatory role [5]. Pri-miRNA is modified by some ways like lysis, transport, and cleavage to produce mature miRNA [6], the latter guide RNA-induced silencing complex to cut the target mRNA domain by complementing the 3′UTR base of the target gene, thus reducing the expression of target mRNA or inhibiting its translation [4]. miRNA is a critical regulator of gene regulation in the biogenesis and can regulate adipocyte differentiation by interacting with target genes [7]. It can directly bind to important transcription factors associated with adipocyte differentiation or participate in signaling pathways that regulate cell differentiation [8]. For instance, miR-27b target lipoprotein lipase and inhibit human adipose-derived stem cells differentiation [9]. In 3T3-L1 culture, miR-425 was found to regulate lipid generation and metabolism by controlling multidirectional targets such as MAPK4/P38 and Cab39/AMPK [10]. Researchers have identified 104 differentially expressed genes from intramuscular adipocytes in late-laying-period hens and juvenile hens by RNA-seq. Integrated analysis of miRNA and genes showed that differentially expressed miRNAs/mRNAs were enriched in pathways such as ubiquitin-mediated proteolysis, the PPAR signaling pathway and other lipid metabolism pathways, and further study proved that gga-miR-140-5p promotes the differentiation of intramuscular adipocytes via targeting retinoid X receptor gamma [11].

Transcriptome sequencing (RNA-seq) is widely used in detecting the overall transcription level of the species. Besides, it is also used to find new transcripts by analyzing the structure and expression level of the enriched single-stranded mRNA, which confirms the results of the expression level of low-abundance genes [12]. Jianzhou Da’er goat is a new breed that is crossed between Jianyang native goat and Nub goat in southwest China, it has the characteristics of fast growth and strong reproductive performance. Therefore, we took subcutaneous adipocytes from Jianzhou da’er goat as the object and cultured *in vitro*, then miRNA-seq was used to explore the expression patterns of miRNA in preadipocytes and adipocytes. Except that, we further characterized the effect of observed miR-133a-3p on the differentiation of goat subcutaneous adipocytes through function gain and function loss experiments. In summary, this study provided a theoretical basis for elucidating the functional mechanism of miRNA-mediated target genes and a reliable mathematical basis for the construction of a goat adipocytes differentiation regulatory network.

## MATERIALS AND METHODS

All experimental procedures were reviewed and approved by the Institutional Animal Care and Use Committee, Southwest Minzu University (Chengdu, Sichuan, China) (No.18032), and all the experiments complied with the requirements of the directory of the Ethical Treatment of Experimental Animals of China.

### Preparation of RNA-seq samples

The preparation of sample is the same as our previous work [13] and pre-adipocytes (0 d) were regarded as the control PC, and mature adipocytes formed after 3 days of culture were regarded as PE (n = 5). Total RNA from preadipocytes and adipocytes was isolated following protocol of TRIzol (Takara, Dalian, China). Nanodrop (IMPLEN, Palo Alto, CA, USA) was used to detect RNA purity and integrity.

### Library preparation for RNA-seq

**Figure 1:**
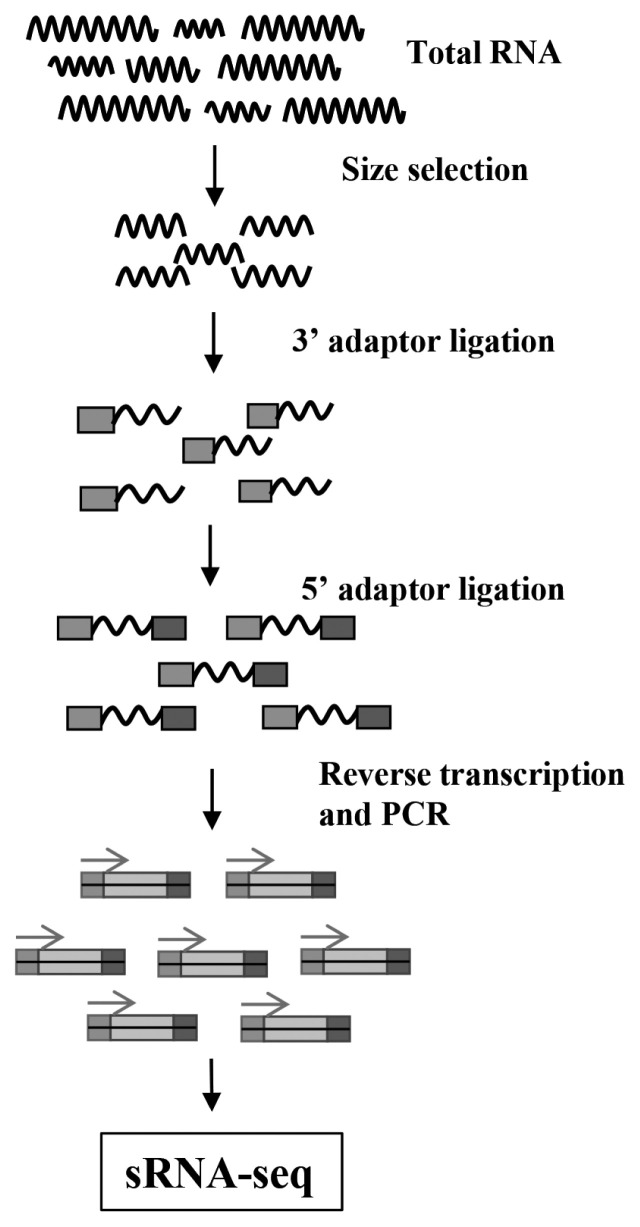
The flow diagram of sRNA-Seq. Total RNA extracted from samples was separated by gel, then the 15–35 nt fragments were selected to connect 3′ adaptor and 5′ adaptor orderly, and these fragments were reverse transcribed and amplified by polymerase chain reaction to complete the small RNA library construction, further sequencing by Illumina HiSeq.

The 15–35 nt RNA fragments were separated and collected by gel, 3′ and 5′ adapters were connected at both ends of the RNA fragments, then the fragments were reverse transcribed into cDNA, amplified by polymerase chain reaction (PCR) to establish a sequencing library. The constructed library was sequenced by Illumina HiSeq. The process of library construction for RNA-seq was as Figure 1.

### Quality evaluation of sequencing results, sequence comparison and quantitative analysis

The raw reads obtained by RNA-Seq were filtered to remove low-quality reads and reads with adapters to obtain clean reads. Qphred = −10 log10 (e) was used to calculate the error rate. Novoaligen was used to compare clean reads with the miRBase database to identify miRNAs, then mirDeep was used to predict novel miRNAs. What’s more, novoaligen and samtools were used for quantitative analysis, and reads per million was used for standardization.

### Screening of differently expression miRNAs, cluster analysis and construction of miRNA-mRNA network

DEGSeq was used to analyze the differentially expressed miRNA, Padjust<0.05 was used to indicate the significance of differentially expressed miRNAs (DEm); DEms were subjected to gene ontology (GO) and Kyoto encyclopedia for genes and genomes (KEGG) enrichment analysis; miRanda was used to predict miRNA target genes.

### miRNAs quantitative polymerase chain reaction verification

Five differentially expressed (DE) miRNAs were selected randomly for quantitative PCR (qPCR) to verify the accuracy of the sequencing results. Taken 1 μg of total RNA for reverse transcription according to the instructions of the Mir-X miRNA First-Strand Synthesis kit (Takara, China), qPCR system and procedures were as the kit instructions. miRNAs 5′ primer information was listed in Table 1, U6 was utilized to normalize miRNA expression, the miRNA 3′ primer and U6 primer were provided by the kit.

### Cell transfection

The F3 goat subcutaneous preadipocytes (12-well plate) were transfected with chemically synthesis chi-miR-133a-3p mimics, inhibitors, and negative control (Genepharma, Shanghai, China) by TurboFect Transfection Reagent (Thermo Fisher Scientific, Waltham, MA, USA) when the cell confluence reached 80%. All operations were in accordance with the instructions. After 14 hours of transfection, added 2 mL 50 μM oleic acid (Sigma, St. Louis, MO, USA) medium to each well to induce differentiation, and the adipocytes were collected after 3 days of culture.

### Bodipy staining

Cultured cells (24-well plate, transfection was same as “Cell Transfection”, reagent consumption halved) were washed with phosphate-buffered saline (PBS) and fixed with 4% formaldehyde for 30 min at room temperature. Then the cells were stained using the Bodipy (Thermo Fisher Scientific, USA) working solutions for 20 min under dark conditions, then the cells were washed 3 times with PBS. The shape and number of lipid droplets were observed under the microscope and take images. And the ImageJ was used to quantify Bodipy staining results.

### Oli red O staining

The operations of oil red O staining were the same as Bodipy. After being photographed, oil red O dye (Solarbio, Beijing, China) was extracted from stained adipocytes with 100% isopropanol (Jinshan, Chengdu, China), and the Oil red signal was quantified by measuring the optical density at 490 nm.

### Quantitative polymerase chain reaction

RNA isolated was the same as “Preparation of RNA-seq Samples”, the efficiency of chi-miR-133a-3p mimics and inhibitor was detected by miRNAs qPCR. mRNA was reverse transcribed by RevertAid First Strand cDNA Synthesis Kit (Thermo Fisher Scientific, USA), and the cDNA was used to detect the genes expression level. Ubiquitously expressed transcript gene (*UXT*) was selected as internal reference gene to normalize the mRNA levels in adipocyte. The qPCR system included TB Green Premix Ex Taq II (2×) (Takara, China) 10 μL, sense/antisense primer 1 μL, cDNA 1 μL, ddH_2_O up to 20 μL, 95°C 3 min, 95°C 10 s, melting-out temperature (TM) 10 s, 72°C 15 s, 40 cycles, primer information was shown as Table 2.

### Data analysis

The qPCR data were analyzed by 2^−ΔΔCt^ method and shown as “Means±standard error of the mean” with Graphpad Prism 8.0 (Version X; La Jolla, CA, USA). The significance of data was analyzed by one-way analysis of variance in SPSS (Version X; IBM, Armonk, NY, USA).

## RESULTS

### Quality evaluation of miRNA-seq data

After removing low-quality reads, about 9.5 to 12.1 million and 9.5 to 11.4 million of clean reads were obtained from the PC and PE, respectively. The error rate of each sample was less than 0.01%, and both Q20 and Q30 were greater than 94% (Table 3). The mapping ratio of the samples in the sRNA library was between 84% to 90% (Table 4). The above data comprehensively indicated the data with high quality, which provided a reliable basis for further analysis.

### Correlation analysis of miRNA-seq and characteristic analysis of miRNAs

**Figure 2:**
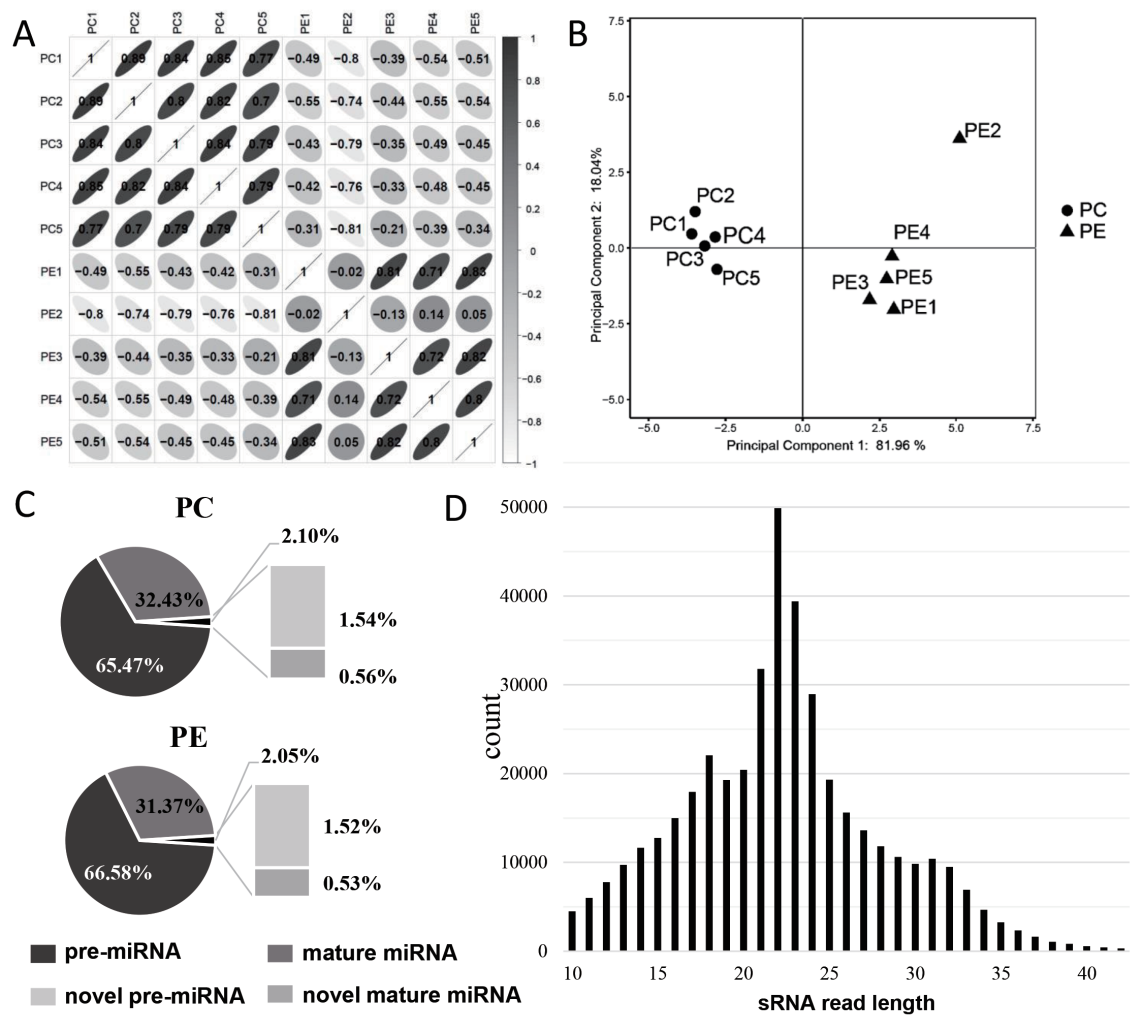
Characteristic analysis of miRNAs. (A) Bar plot of correlation coefficient analysis, the darker of the color means the greater the correlation coefficient, Pearson correlation coefficient (r) was showed in the box, r<0 represents negative correlation and r>0 represents positive correlation. (B) PCA analysis between PC and PE, PE2 showed a large distance from other samples in the PE group, and we excluded PE2 data in later analysis. (C) The composition of miRNA in PC and PE group and the ratio of each component. (D) Length distribution map of miRNAs. PCA, principal component analysis; PC, preadipocytes; PE, mature adipocytes by adipogenic differentiation.

Correlation analysis of the RNA-seq samples showed that the samples were clearly divided into two groups, and the correlation coefficient of the samples in each group ranged from 0.952 to 0.995 (Figure 2A), but the correlation between PE2 and other samples was relatively low. The results of principal component analysis (PCA) showed that the samples were well correlated, however, PE2 was separated from PE group (Figure 2B). Therefore, the data of PE2 were excluded from subsequent sequencing data analysis to avoid affecting the accuracy of the data.

The reads in the sRNA library were analyzed, and it was found that about 98% of the reads in PC and PE were mapped to the annotated miRNA, and pre-miRNA accounted for a higher proportion in either annotated miRNA or novel miRNA (Figure 2C). The lengths of the screened miRNAs were analyzed, and we found that the miRNAs were mainly concentrated in 21 to 24 nt, about 35.73%; and the miRNAs with lengths less than 18 nt, 18 to 24 nt, and more than 24 nt were 20.32%, 50.44%, and 29.24%, respectively (Figure 2D).

### Screening of differentially expressed miRNAs in goat subcutaneous adipocytes

**Figure 3:**
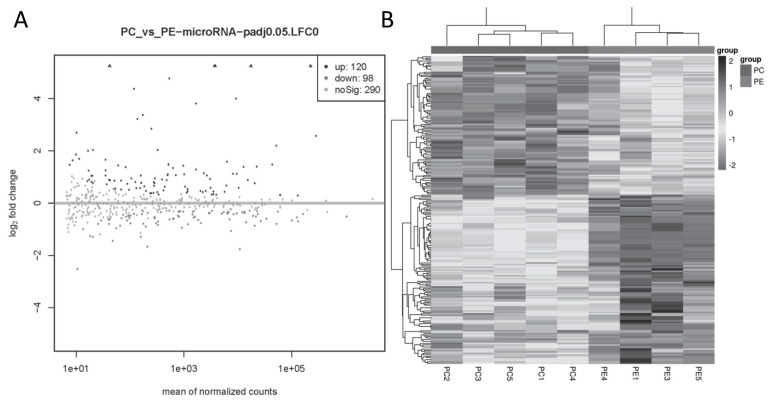
DE miRNA in goat subcutaneous preadipocytes and adipocytes. (A) Volcano plot of DE miRNAs, |log2FoldChange|>0 and q value<0.05 as the threshold, log2FoldChange>0, q value<0.05 represents the up-regulated miRNA and shown as black dots, while log2FoldChange<0, q value<0.05 represents the down-regulated miRNA and shown as dark grey dots, and light gray dots means the miRNA with no significant. (B) Heatmap of DE miRNAs, the black box represents higher expression, and the grey box represents lower expression, each horizontal represents a DE miRNA. DE, differentially expressed.

A total of 218 miRNAs were DE in the PC and PE group, of which 120 were up-regulated (89 annotated and 31 novel miRNAs) and 98 were down-regulated (67 annotated and 31 novel miRNAs) (Figure 3A). The cluster analysis of DE miRNAs found that the samples were clearly clustered into two categories (Figure 3B).

### Analysis of GO and KEGG

**Figure 4:**
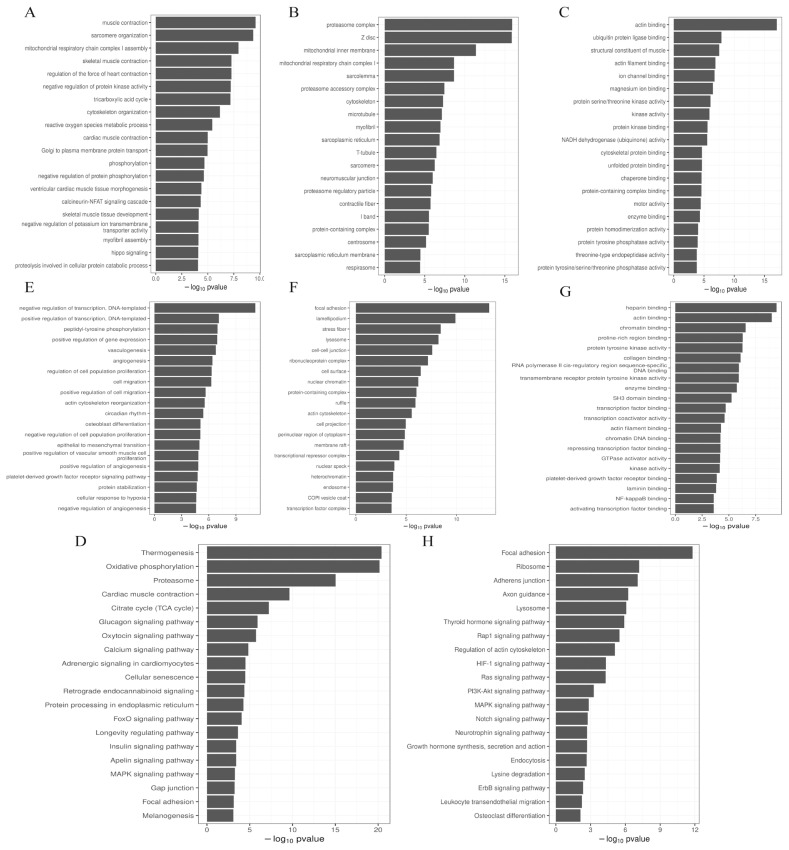
GO and KEGG analysis of DE miRNAs target genes. (A) GO terms in BP enriched by up-regulated miRNAs target genes. (B) GO terms in CC enriched by up-regulated miRNAs target genes. (C) GO terms in MF. (D) enriched by up-regulated miRNAs target genes. (D) Up-regulated miRNAs target genes are enriched in the KEGG pathway. (E) Down-regulated miRNAs target genes are enriched in GO terms in BP. (F) Down-regulated miRNAs target genes are enriched in GO terms in CC. (G) Down-regulated miRNAs targets gene-enriched GO terms in MF. (H) KEGG pathway enriched by up-regulated miRNAs target genes. GO, gene ontology; KEGG, Kyoto encyclopedia for genes and genomes; DE, differentially expressed; BP, biological process; CC, cellular component; MF, molecular function.

The prediction of the DE miRNA target genes was by GOseq, and perform GO enrichment analysis on the target genes, we found that the most enriched biological process (BP) (Figure 4A), cellular component (CC) (Figure 4B) and molecular function (MF) (Figure 4C) term of the target genes of up-regulated miRNAs were muscle contraction, proteasome complex and actin binding, respectively. The most significantly enriched BP (Figure 4E), CC (Figure 4F), and MF (Figure 4G) term for the target genes of down-regulated miRNAs were negative regulation of transcription (DNA-templated), focal adhesion, and heparin binding, respectively. KEGG enrichment analysis found that the common enrichment pathways for up-regulated and down-regulated miRNAs were Focal adhesion and MAPK signaling pathway. In addition, the up-regulated miRNAs were most enriched in the Thermogenesis pathway, the Oxytocin signaling pathway, FoxO signaling pathway, and insulin signaling pathway were also significantly enriched (Figure 4D). The down-regulated miRNAs were most significantly enriched in the Focal adhesion pathway, and the PI3K-Akt signaling pathway and Notch signaling pathway were also enriched (Figure 4H).

### qPCR validation

**Figure 5:**
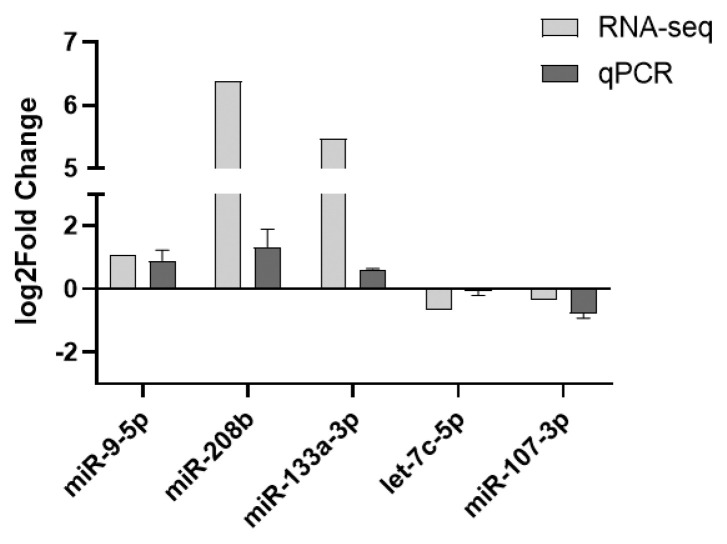
Validating the expression trends of the 5 differentially expressed miRNAs by quantitative reverse-transcription polymerase chain reaction (qPCR). The log2Foldchange>0 indicates that the miRNA is up-regulated, while log2Foldchange<0 indicates that the miRNA is down-regulated. The qPCR results showed the same trend with RNA-seq, indicating the RNA-seq data reliable.

Five DE miRNAs were chosen randomly to verify the expression level by qPCR, the result showed that the expression trend of miRNAs by qPCR was similar to miRNA-seq (Figure 5).

### miR-133a-3p promotes adipocytes differentiation in goat

**Figure 6:**
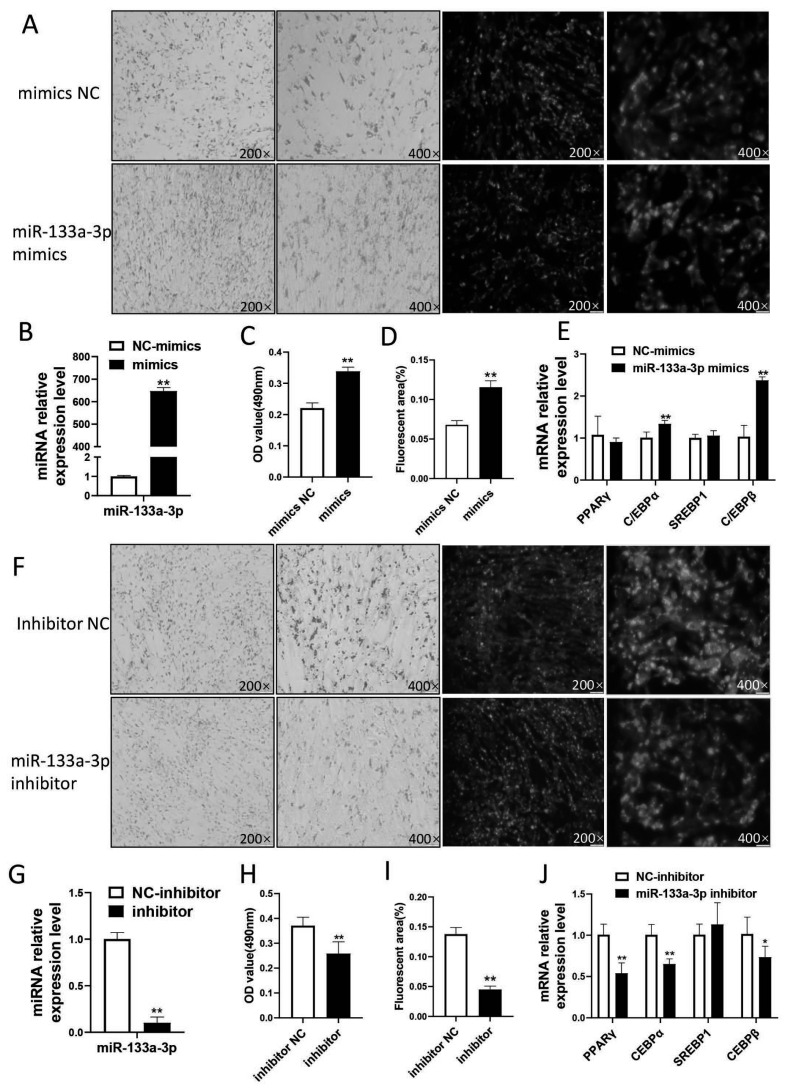
The effect of miR-133a-3p in goat subcutaneous adipocytes differentiation (A), (F): Lipid droplet accumulation after simulating/inhibiting miR-133a-3p expression by oil red O and BODIPY staining. (B), (G): efficiency of miR-133a-3p overexpression/interference. (C), (H): the OD value by oil red O staining. (D), (I): fluorescence quantification by BODIPY staining. (E), (J): changes in the expression of marker genes by overexpression/inhibition of 133. * means p<0.05, ** means p<0.01. 3 biological replicates were set for each treatment group.

miR-133a-3p was selected for functional verification in further study. We firstly constructed an overexpression model by transfecting subcutaneous pre-adipocytes with miR-133a-3p mimics. The result showed that the expression of miR-133a-3p was up-regulated 647 times in subcutaneous pre-adipocytes that transfected with miR-133a-3p mimics when compared to the control (Figure 6B). Oil red O staining and BODIPY staining showed that the accumulation of lipid droplets in the mimics group was greater than that in the NC group (p<0.01) (Figure 6A,C,D). Besides, we found that the differentiation marker gene CCAAT enhancer binding protein α (*C/EBPα*) and *C/EBPβ* was significantly increased in miR-133a-3p mimics (p<0.01) (Figure 6E).

Then we conducted a miR-133a-3p loss model by transfecting with miR-133a-3p inhibitor. After transfection of miR-133a-3p inhibitor, the expression of miR-133a-3p was down-regulated about 90% (Figure 6G). Oil red O staining and BODIPY staining showed that the lipid droplet accumulation in the inhibitor group was significantly less than that in the NC group (p<0.01 (Figure 6F,H,L)). The expression level of differentiation marker genes peroxisome proliferator activated receptor gamma (*PPARγ*) and *C/EBPα* was extremely significantly reduced (p<0.01), and *C/EBPβ* was significantly reduced (p<0.05) (Figure 6J).

## DISCUSSION

miRNAs played a regulatory role in many biological processes, including adipocyte differentiation and lipid metabolism [14]. The regulatory network of miRNAs was extremely complicated because one miRNA could target multiple different mRNAs. Adipocyte differentiation was the main source of fat deposit, but the regulatory role of most miRNAs in goat subcutaneous adipocyte differentiation was still unclear. RNA-seq technology has been widely used in different samples to screen key mRNAs and non-coding RNAs [15] or key signaling pathways [16]. Therefore, miRNA-seq was used in this study to obtain key miRNAs in the differentiation of goat subcutaneous adipocytes to understand molecular mechanism in goat fat deposition. As a result, differently expressed miRNAs and several pathways enriched in regulating the differentiation of adipocytes were identified through functional analyses.

From both the aspect of cell sample morphology observation and sequencing quality evaluation results, it was shown that the sequencing samples with high quality, the sequencing results with high utilization rate. We found that the length of miRNA was mainly distributed in 21 to 24 bp, which is in line with the length of mammalian miRNA sequence [4]. In this study, using |log2FoldChange|>0 and q value<0.05 as the thresholds, 218 differentially expressed miRNAs were screened, of which 120 were up-regulated and 98 were down-regulated. Among them, the five miRNAs with the largest up-regulation changes were miR-208b, miR-499-5p, miR-133a-5p, miR-133a-3p and miR-1, and the five miRNAs with the largest down-regulation changes were miR-335-3p, let-7c-3p, miR-196a, miR-16b-5p, and miR-15b-5p. It has been reported that finishing pigs fed a diet containing 0.3% butyrate had a higher intramuscular fat content than the pigs fed a basal diet, the fat deposits associated with this process resulted in a decrease in the expression level of miR-133a-3p and an increase in expression of miR-208b and miR-499-5p [17]. miR-499-5p could target PTEN, which in turn affected the PI3K/AKT/GSK signaling pathway and glycogen synthesis [18]. miR-133a could inhibit the browning of adipocytes [19], which may partly decrease adipocyte loss. Inhibition of miR-499-5p expression in mice with nonalcoholic fatty liver could reduce the accumulation of lipid and triglyceride and reduce total cholesterol in serum in individual mice [20]. miR-133a-5p was down-regulated in subcutaneous fat deposition in rats fed high-fat diet (HFD), but up-regulated in HFD mice [21]. Overexpression of miR-196a induces preadipocyte differentiation by increasing the expression of adipocyte-specific markers, lipid accumulation and triglyceride content [22]. miR-196a could mediate the conversion of white adipocyte progenitor cells to brown adipocytes through Hoxc8-C/EBPβ [23]. In C2C12 cell adipogenesis induced by cocktail, miR-15b-5p was down-regulated, it targeted Akt3 to stimulate adipogenic differentiation [24]. The above studies have shown that DE miRNAs also play an important role in the differentiation of goat white adipocytes.

The DE miRNA target genes were predicted, and enrichment analysis was performed. KEGG enrichment analysis showed that the common enrichment pathways for up-regulated and down-regulated miRNAs were focal adhesion kinase (FAK) and MAPK signaling pathway. Interestingly, Thermogenesis pathway was most significant enriched by up-regulate miRNAs, and the FAK pathway was most significant enriched by down-regulate miRNAs. Studies have shown that the FAK participated in adipocyte differentiation, and its cleavage by calpain was required to fulfill the final maturation of adipocytes [25]. Study had also shown that liver kinase B1 (LKB1) inhibited goat intramuscular adipogenesis through the FAK pathway, blocking this pathway rescued the observed phenotypes in LKB1 knockdown adipocytes [26]. MAPK phosphatase-1 (MKP-1) plays an essential role in adipocyte differentiation through down-regulation of p42/p44 MAPK activity [27]. C1q/TNF-related protein 6 (CTRP6) promotes porcine intramuscular and subcutaneous adipocyte differentiation through the MAPK signaling pathway [28]. Interleukin-27 (IL-27) promoted adipocyte thermogenesis and energy expenditure by activating p38 MAPK-PGC-1α signaling and stimulating uncoupling protein 1 (UCP1) [29].

Adipose tissue acts against cold through thermogenesis. Thermogenesis is initiated physiologically by cold sensation or dietary intake, which stimulates sympathetic release of norepinephrine to activate β3-adrenergic receptors (β3-ARs) in adipose tissue [30,31]. This pathway promotes thermogenesis through a protein kinase A (PKA)-p38 MAPK-Ucp1 signaling axis [32,33]. Besides, pathways such as the calcium signaling pathway, FoxO signaling pathway, and insulin signaling pathway PI3K-Akt signaling pathway and Notch signaling pathway were also significantly enriched. Numerous studies have confirmed that the above-mentioned enriched pathways were involved in the differentiation process of adipocytes. For instance, Calcium signaling pathway was a key pathway in regulating obesity [34], Ca2+ promote adipocyte differentiation and metabolism [35], the PI3K/Akt signaling pathway could increase lipid accumulation [36] and promote cell adipogenic differentiation [37]. Both FoxO signaling pathway [38], insulin signaling pathway [39] and Notch signaling pathway [40] have been reported to be involved in adipocyte adipogenesis. These signaling pathways can not only function independently, but they also interact with and regulate each other, for example, in adipocytes, the activation of p38-MAPK signaling pathway can promote intracellular calcium transport to regulate adipocytes metabolism to reduce obesity [41,42]. Therefore, the enriched pathways of DEm are mostly related to adipocyte differentiation, this work will provide support for further studies revealing the regulatory network during goat adipocyte differentiation.

We selected miR-133a-3p as the object for further research. We found that simulating its expression in preadipocytes could promote the differentiation of subcutaneous adipocytes, while inhibiting its expression inhibited their differentiation, indicating that miR-133a-3p was a positive regulator of subcutaneous adipocyte differentiation. Studies had reported that the expression of miR-133a-3p was low in the subcutaneous adipose tissue of obese patients, and its expression was increased in individuals who lose weight after energy restriction, suggesting that miR-133a-3p seems to be a negative regulator of adipogenesis [43]. This was inconsistent with the results of our study, presumably due to different experimental subjects. So, how miR-133a-3p regulates goat subcutaneous adipocyte adipogenesis still needs further study.

## CONCLUSION

This study identified 218 differentially expressed miRNAs between pre-adipocytes and mature adipocytes in goat subcutaneous fat. The pathway enrichment analysis of DE miRNA target genes showed that pathways related to adipocyte differentiation and fatty acid metabolism were enriched. Furthermore, we found that miR-133a-3p positively regulate the differentiation of goat adipocytes.

## Figures and Tables

**Table 1:** miRNAs 5′ primer information

miRNA	5′ primer sequence
chi-miR-9-5p	TCTTTGGTTATCTAGCTGTATGA
chi-miR-208b	TATAAGACGAACAAAAGGTTTG
chi-miR-133a-3p	TTTGGTCCCCTTCAACCAGCTGT
chi-let-7c-3p	CTGTACAACCTTCTAGCTTTCC
chi-miR-107-3p	AGCAGCATTGTACAGGGCTAT

**Table 2:** qPCR primer information for genes

Primer's name	Sequence	TM (°C)	Product length/bp	Genbank
*PPARγ*	AAGCGTCAGGGTTCCACTATG	60	197	NM_001285658
	GAACCTGATGGCGTTATGAGAC			
*C/EBPα*	CCGTGGACAAGAACAGCAAC	58	142	XM_018062278
	AGGCGGTCATTGTCACTGGT			
*C/EBPβ*	CAAGAAGACGGTGGACAAGC	66	204	XM_018058020.1
	AACAAGTTCCGCAGGGTG			
*SREBP1*	AAGTGGTGGGCCTCTCTGA	58	127	NM_001285755
	GCAGGGGTTTCTCGGACT			
*UXT*	GCAAGTGGATTTGGGCTGTAAC	60	180	XM_005700842.2
	ATGGAGTCCTTGGTGAGGTTGT			

qPCR, quantitative reverse-transcription polymerase chain reaction; TM, melting-out temperature; *PPARγ*, peroxisome proliferator-activated receptor γ; *C/EBPα*, CCAAT enhancer binding protein α; *C/EBPβ*, CCAAT enhancer binding protein β; *SREBP1*, Sterol-regulatory element binding protein 1; *UXT*, ubiquitously expressed transcript gene.

**Table 3:** Quality assessment of sequencing data

Sample names	Raw reads	Clean reads	Raw bases (G)	Clean bases (G)	Error rate (%)	Q20 (%)	Q30 (%)	GC content (%)
PC1	11,598,661	11,559,615	0.58	0.578	0.01	97.41	94.97	48.34
PC2	11,918,096	11,875,275	0.596	0.594	0.01	97.41	94.99	48.31
PC3	10,746,726	10,710,364	0.537	0.536	0.01	97.49	95.13	48.4
PC4	12,126,657	12,083,074	0.606	0.604	0.01	97.44	95.06	48.44
PC5	9,573,538	9,523,390	0.479	0.476	0.01	96.96	94.25	48.35
PE1	11,305,495	11,251,425	0.565	0.563	0.01	97.09	94.48	48.53
PE2	11,380,888	11,334,404	0.569	0.567	0.01	97.23	94.69	47.6
PE3	10,050,601	9,999,778	0.503	0.500	0.01	97.08	94.48	48.53
PE4	10,036,565	9,984,937	0.502	0.499	0.01	97.00	94.37	47.93
PE5	11,427,762	11,367,340	0.571	0.568	0.01	96.97	94.32	48.19

Raw reads: count the original sequence data.

Bases: multiply the number of sequencing sequences by the length of the sequencing sequence and convert it to G as the unit.

Error rate: refers to the sequencing error rate.

Q20: the percentage of bases with a Phred value greater than 20 to the total base.

Q30: the percentage of bases with a Phred value greater than 30 to the total bases.

GC content: calculate the percentage of the total number of bases G and C to the total number of bases.

**Table 4:** Result of sRNA sequence reference genome comparison

Sample name	Total. collapsed	Map. collapsed (ratio)	Unmap. collapsed (ratio)	Total	Map (ratio)	Unmap (ratio)
PC1	414,235	182,358 (44.02%)	231,877 (55.98%)	11,190,097	9,760,858 (87.22%)	1,429,239 (12.78%)
PC2	424,272	189,827 (44.74%)	234,445 (55.26%)	11,569,005	10,080,583 (87.13%)	1,488,422 (12.87%)
PC3	417,804	193,543 (46.32%)	224,261 (53.68%)	10,375,373	9,016,441 (86.90%)	1,358,932 (13.10%)
PC4	447,476	185,803 (41.52%)	261,673 (58.48%)	11,690,267	10,131,181 (86.66%)	1,559,086 (13.34%)
PC5	362,507	155,189 (42.81%)	207,318 (57.19%)	9,177,248	8,033,435(87.54%)	1,143,813 (12.46%)
PE1	474,389	195,068 (41.12%)	279,321 (58.88%)	10,846,145	9,194,795(84.77%)	1,651,350 (15.23%)
PE2	318,225	123,914 (38.94%)	194,311 (61.06%)	11,055,221	9,852,386 (89.12%)	1,202,835 (10.88%)
PE3	436,123	175,940 (40.34%)	260,183 (59.66%)	9,659,473	8,134,747 (84.22%)	1,524,726 (15.78%)
PE4	361,622	167,085 (46.20%)	194,537 (53.80%)	9,631,449	8,598,224(89.27%)	1,033,225 (10.73%)
PE5	440,224	192,546 (43.74%)	247,678 (56.26%)	11,033,415	9,620,577(87.19%)	1,412,838 (12.81%)
